# Supplementary figures and images for: Validation of the Unesp-Botucatu pig composite acute pain scale (UPAPS) in piglets undergoing castration
Source: PLoS One. 2023 Apr 13;18(4):e0284218. doi: 10.1371/journal.pone.0284218 (PMC10101451; doi:10.1371/journal.pone.0284218)

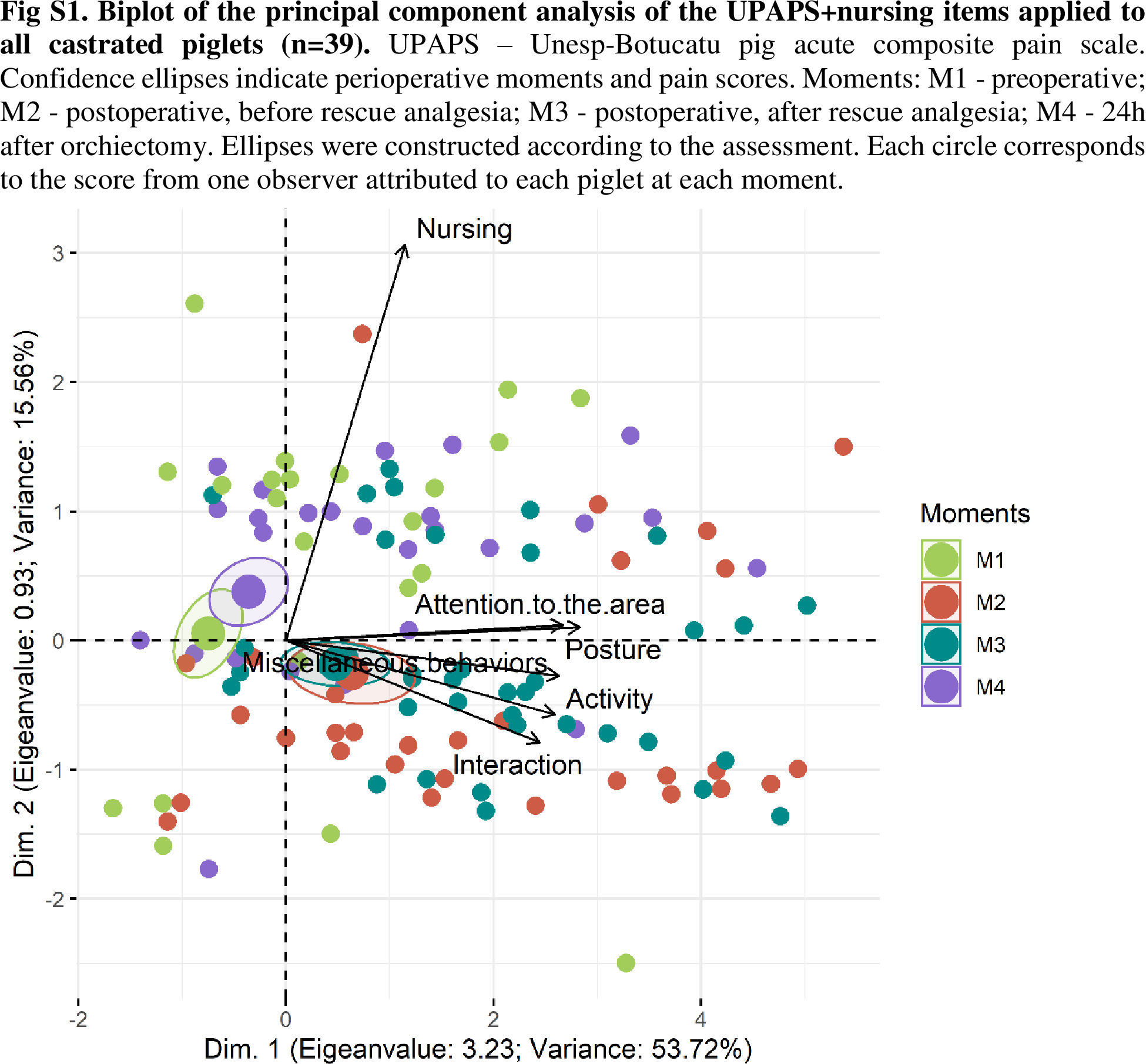

Supplement: S1 Fig — UPAPS–Unesp-Botucatu pig acute composite pain scale. Confidence ellipses indicate perioperative moments and pain scores. Moments: M1—preoperative; M2—postoperative, before rescue analgesia; M3—postoperative, after rescue analgesia; M4 - 24h after orchiectomy. Ellipses were constructed according to the assessment. Each circle corresponds to the score from one observer attributed to each piglet at each moment. (TIF) [file pone.0284218.s001.tif]

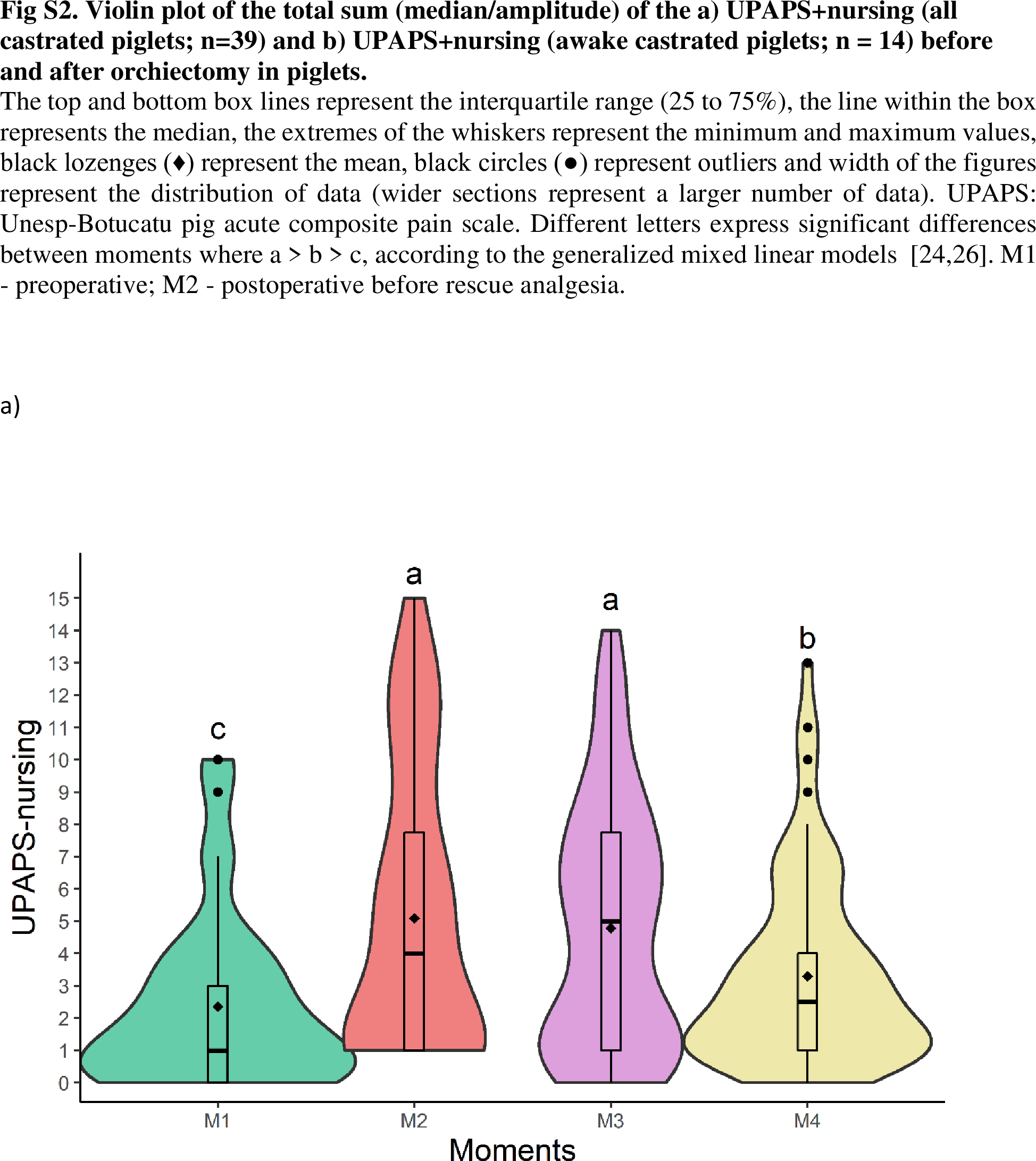

Supplement: S2 Fig — The top and bottom box lines represent the interquartile range (25 to 75%), the line within the box represents the median, the extremes of the whiskers represent the minimum and maximum values, black lozenges (♦) represent the mean, black circles (●) represent outliers and width of the figures represent the distribution of data (wider sections represent a larger number of data). UPAPS: Unesp-Botucatu pig acute composite pain scale. Different letters express significant differences between moments where a > b > c, according to the generalized mixed linear models [24,26]. M1—preoperative; M2—postoperative before rescue analgesia. (TIF) [file pone.0284218.s002.tif]

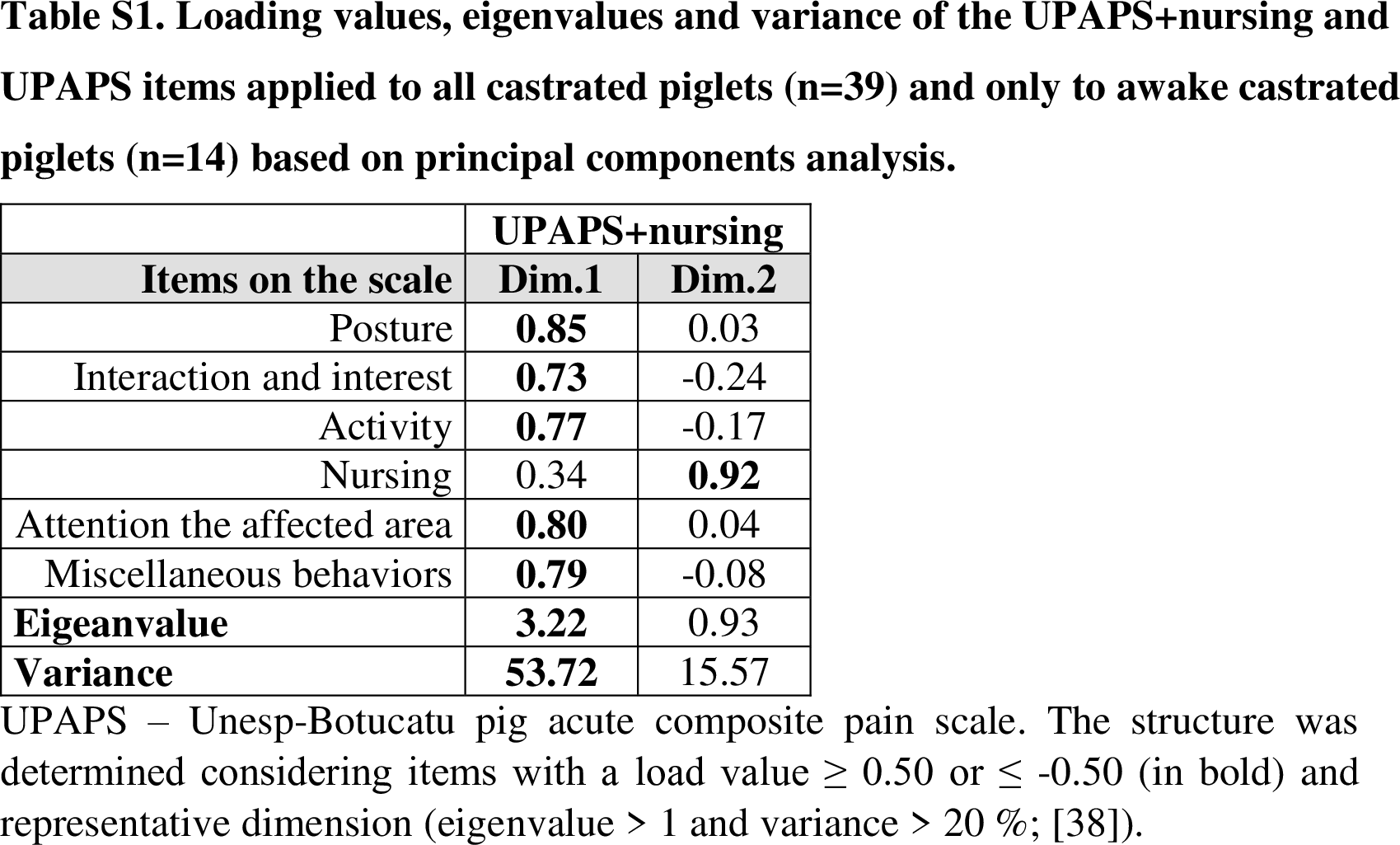

Supplement: S1 Table — (TIF) [file pone.0284218.s003.tif]

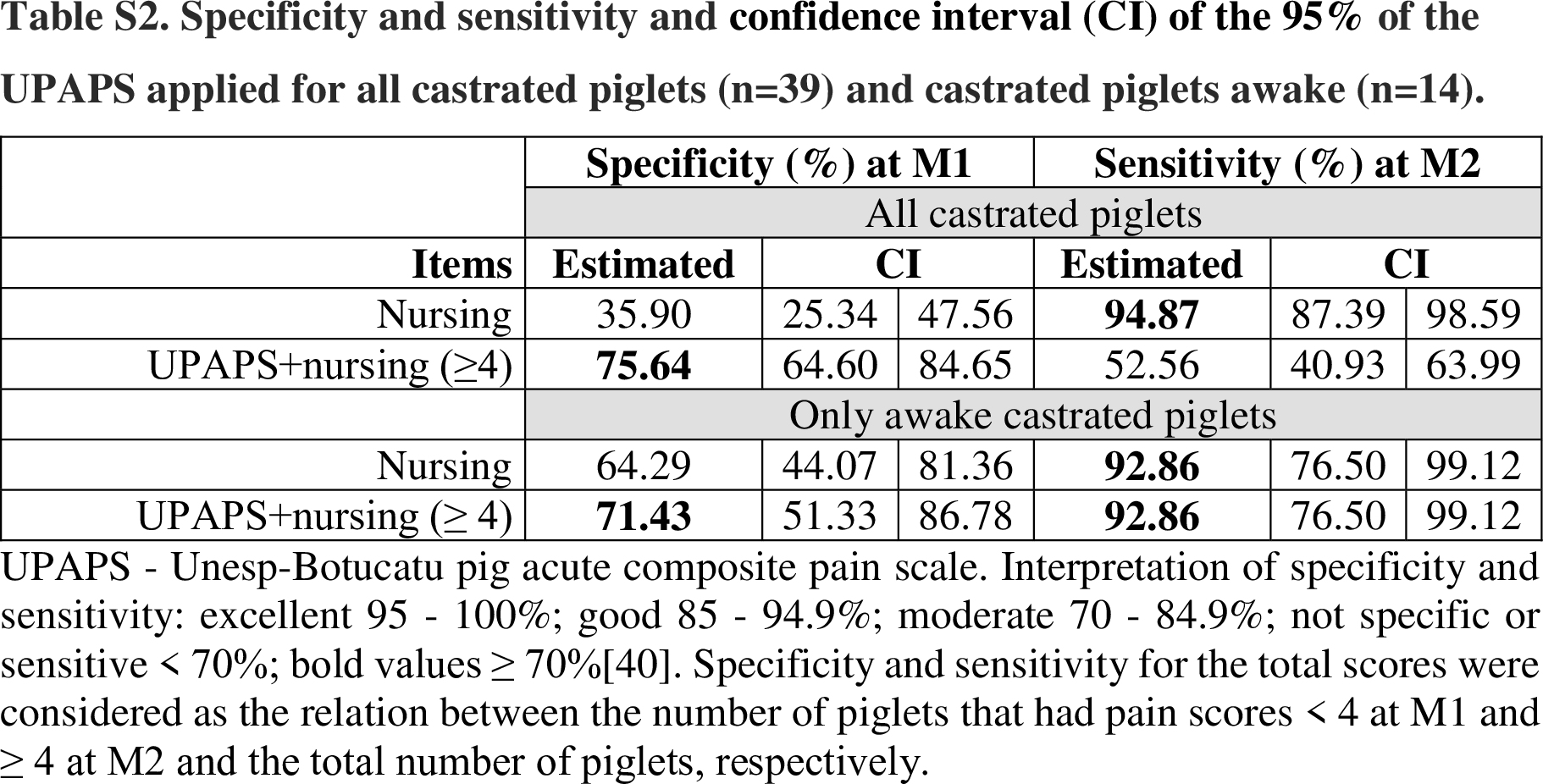

Supplement: S2 Table — (TIF) [file pone.0284218.s004.tif]

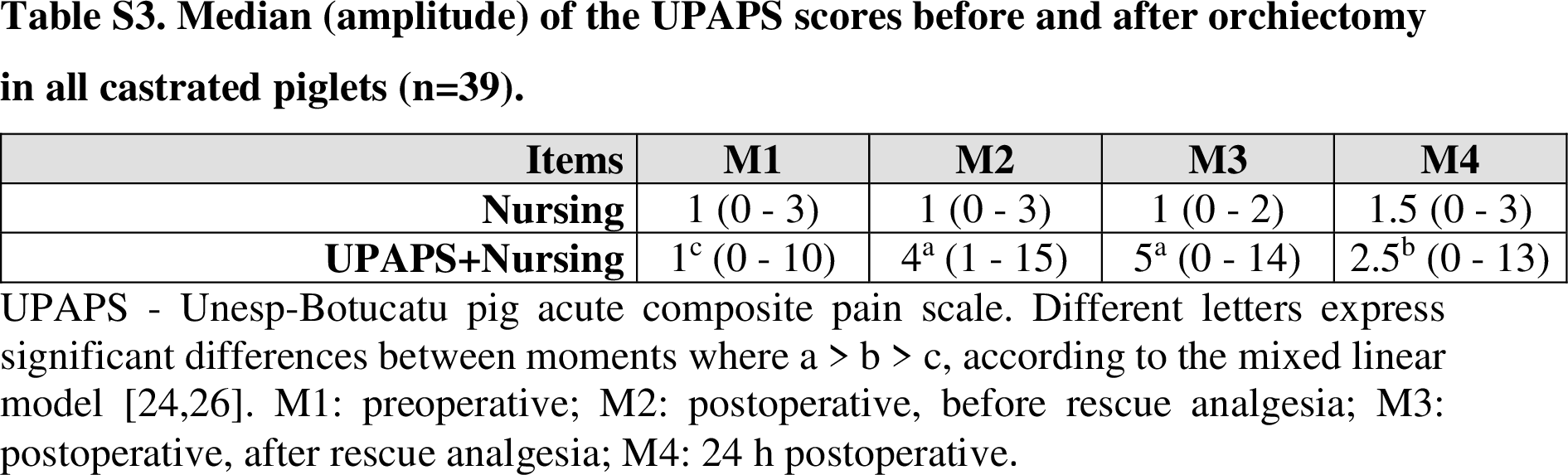

Supplement: S3 Table — (TIF) [file pone.0284218.s005.tif]

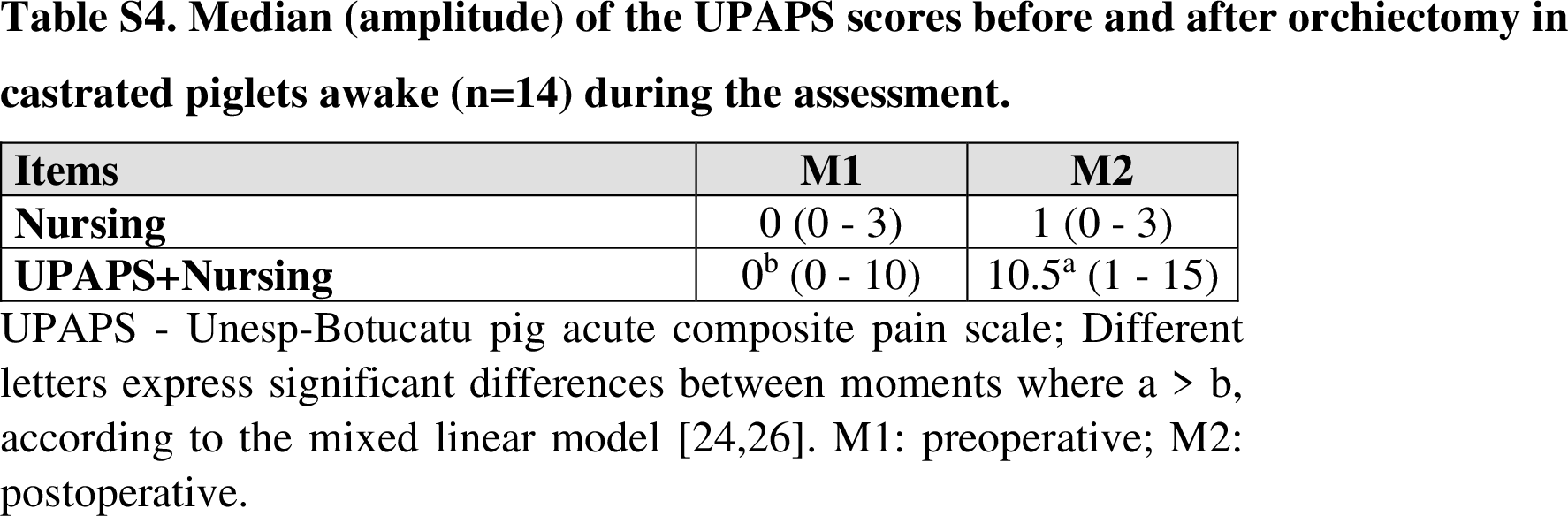

Supplement: S4 Table — (TIF) [file pone.0284218.s006.tif]

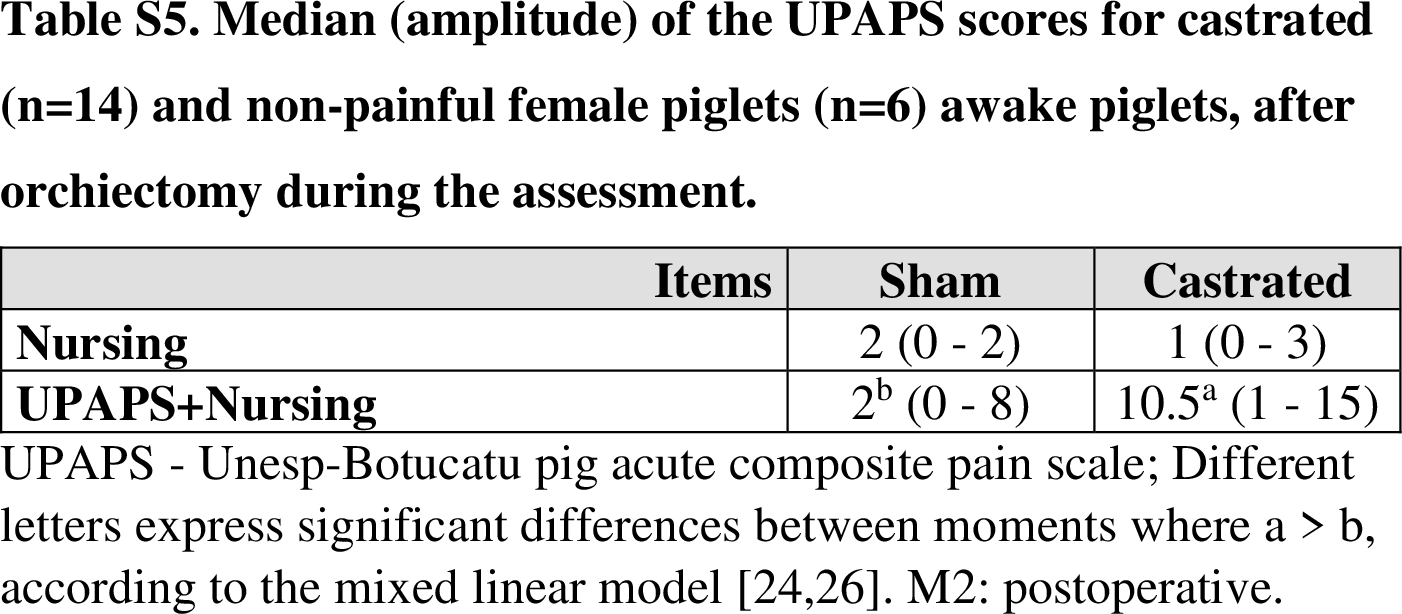

Supplement: S5 Table — (TIF) [file pone.0284218.s007.tif]

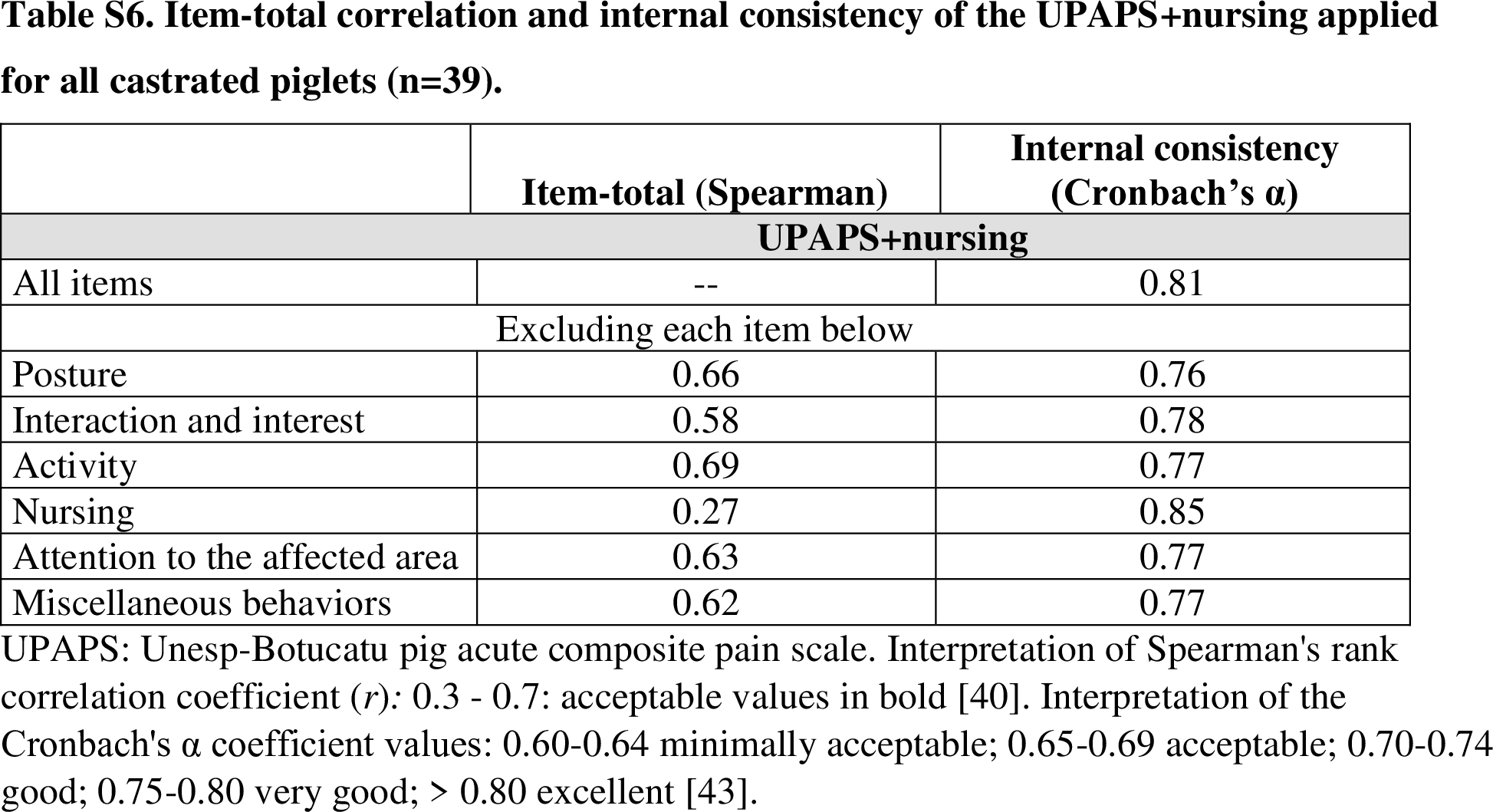

Supplement: S6 Table — (TIF) [file pone.0284218.s008.tif]

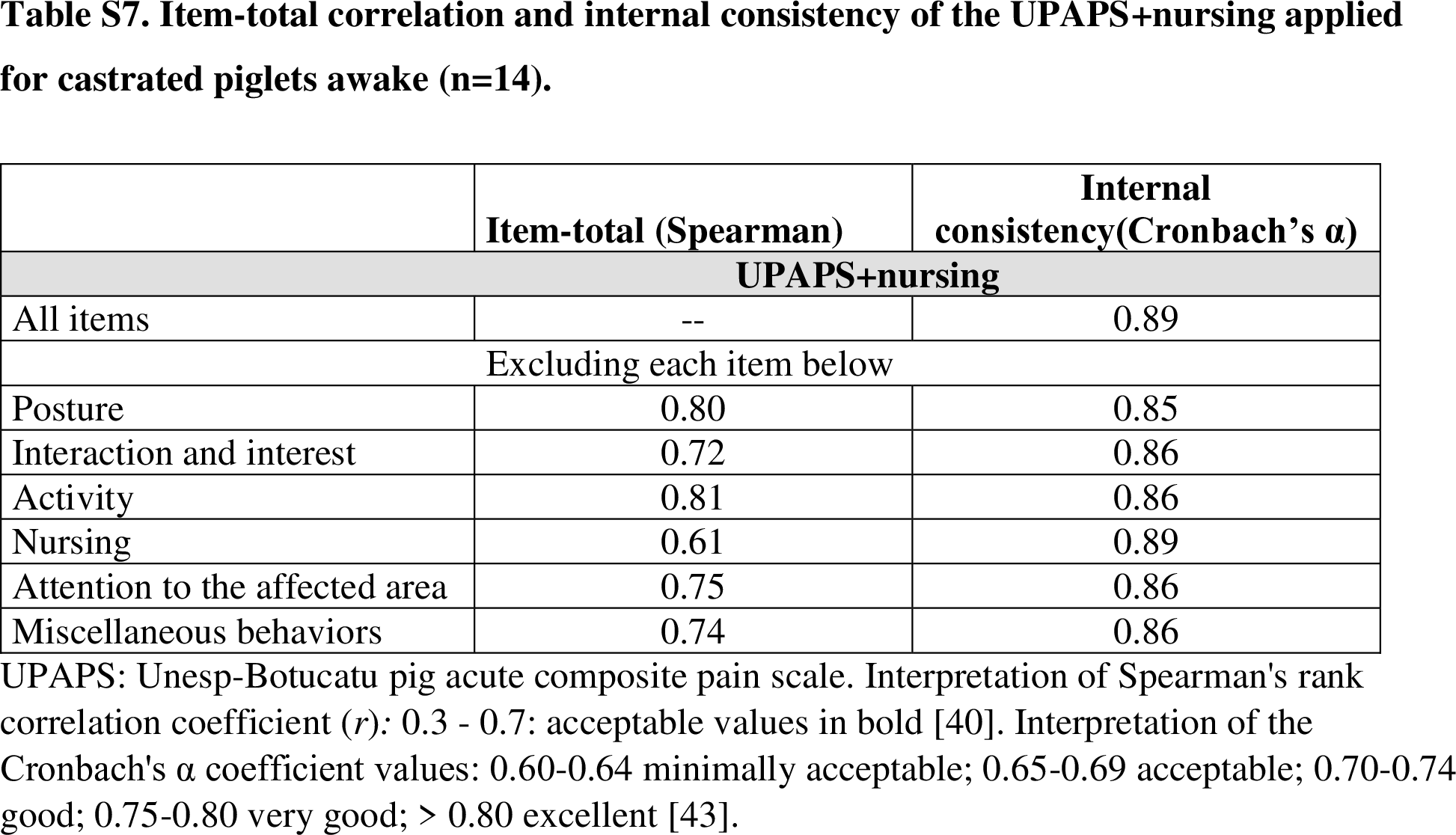

Supplement: S7 Table — (TIF) [file pone.0284218.s009.tif]
