# Supplementary material for: Validation of the Unesp-Botucatu pig composite acute pain scale (UPAPS) in piglets undergoing castration
Source: PLoS One. 2023 Apr 13;18(4):e0284218. doi: 10.1371/journal.pone.0284218 (PMC10101451; doi:10.1371/journal.pone.0284218)
Supplement: Attachment — Submitted filename: Response to reviewers FINAL.docx [file pone.0284218.s011.docx]

Response to reviewers:

Reviewer #1: The authors describe the adaptation of a pain assessment scale (UPAPS) for new-born piglets.

They found the scale functioned well. While it was disappointing that, the scale failed to detect the effects of rescue analgesia. However, this was most likely because the doses rate of flunixin was insufficient for pain control, so this does not detract from the study findings. The scale was found to have good to very good consistency both within and between scorers, so has excellent potential to be developed into a live assessment tool on commercial pig farms and therefore refine the welfare of many animals. The paper should be publishable once the authors have dealt with the following issue, some trivial, others more substantial. The queries below were constructed whilst reading the manuscript, so in some instances the authors have dealt with these at subsequent points in the manuscript. Nevertheless, they should amend the manuscript at the lines indicated to improve readability and comprehension. Note that although the authors state that all data have been made available, this is not technically correct. They say all relevant data are available in the manuscript and supporting information, but the supporting information contains results file, not original data.

**Response:** Thank you for your time and effort in reviewing the manuscript. All comments have been reviewed and responded to accordingly

Line 39: What does the adapted scale mean. Presumably this is described in the main text but should be define in the abstract if mentioned.
The abstract does not adequately describe what behaviours were measured. Sum scores is mentioned - what scores ? The purpose of an abstract is so a reader can quickly discover what the study is about without having to refer to the main text.

**Response**: The abstract has been revised to clarify the adapted scale and include further description of the behavioral items measured

Line 66: Clarify here. The PGS has been shown to effective as indicated in citation 16. Do you mean it has not been validated in terms of being shown to reduce following drug treatment ?

**Response:** Sentence restructured to clarify PGS work

Line 68: add 'the' before development

**Response:** Added

Line 69: insert 'the' before pain literature
**Response:** Added

Line 93: Did the stats control for litter?
**Response:** Original statistical analysis did not include litter as an effect. Analysis was rerun to include litter as a random effect in the model for responsiveness to control for this effect in our sampling. The addition of litter as a random effect resulted in minimal changes that have been addressed in the results and Tables 7, 8, and 9.

Line 95: It’s nice to have example videos to look at but I am not sure how the piglets could be individually identified based on the marks that are visible. Are the example videos the ones that were scored?

**Response**: Example videos include both videos that were scored for this experiment and additional videos that were used for another project. Piglets were identified by either the markings on their back or the color ear tag. For example, posture 0 example is pointing at the pig with the black line on its back, followed by the piglet with the black open circle on the back and lastly the piglet with the purple ear tag. No markings were repeated within a litter and no color was repeated in a litter for pigs evaluated for behavior.

Line 104: What began the day they farrowed until weaning - access to water and feed? I don't think so

**Response:** Statement revised for clarification

Line 106: Personnel is plural and there was only one 'person'
**Response:** Changed to person

Line 124: As females were used as controls - how were the assessors blinded to procedure? More importantly, as behaviour was recorded and scored before castration, the piglets served as their own controls, so why use any females at all?

**Response:** Thank you for the response. We agree that the use of the term “control” for the female piglets is misleading and has been re-written as follows: Female piglets served to assess the effect of sex, handling and social dynamics on the pain scale and were not considered true controls. In addition, video cameras were mounted 2.4m above the ground making it difficulty to visually observer piglet sex in video. However, there is still the possibility that sex was identified on camera and has now been clarified.

Line 126: If so why does the piglet shown in this video have an ear tag and the others don’t?
**Response:** Please see previous response on videos. Videos with piglets with ear tags was collected from another study and was used solely as an example of the behavior. All piglets enrolled in this current study had markings located on their back for identification

Line 133: Pigs were recorded not video.
**Response:** Corrected

Line 136: Why define the recording periods as M1, M2 etc when T1 and T2 would seem more logical? Better Still, to improve readability, why not use B (Baseline), PO (Post-op), PR (Post rescue) and 24h.

**Response:** Thank you for your comments. I agree that the use of different acronyms may be more intuitive, recording periods have been named to maintain consistency with previous work on different species-pain scales to allow for more effective comparison of the work. The authors choose to keep the recording period names

Line 138: Why only 4-minute clips when you have an hours’ worth of data. Seems a waste.

**Response:** 4-min clips were selected based on previous work conducted in pain scale research in livestock (Luna et al, 2020). Additionally, one objective of this work is to identify alternative methodologies to quantify pain sensitivity while minimizing labor, equipment and time requirements for observing behavior. Thus, the selection of 4-min was also conducted to evaluate if pig pain can be detected in shorter behavior observations timepoints

Line 144: As above - how did you blind between males and females. The scrotal sack could be visible in males.

**Response:** the authors agree and have included information stating the potential bias that may have occurred. However, camera angle and height made it very difficult to discern piglet sex so bias, although possible, would be limited.

Line 146: Please explain what is meant here. Do you mean because you only chose one 4 minute clip? Focal sampling would normally mean selecting random segments from the available footage.

**Response**: Agreed. Sentence has been re-written to emphasize that only one piglet was observed for each observation, not multiple piglets at one observation period.

Line 168: This is a repeat of an earlier statement and is not needed, just describe the adaptations not the reason for them.

**Response:** Statement removed

174: Does this mean only awake piglets were scored - it seems obvious it would be, therefore how did that affect numbers scored at each time-point?

**Response**: Paragraph revised to clarify. Awake and asleep piglets were assessed. Piglet state was evaluated at the start of each observation. Piglets were categorized in one of two states: 1) Awake or 2) asleep. Piglet state was then used in the statistical model to to facilitate the discrimination between a piglet sleeping (classified as normal) compared to an awake piglet expressing normal non-painful behavior

Lines 171-180: This whole section is quite confusing. in some instances of sleeping, they were scored as 2, but for pain scoring they were scored 0 when sleeping.
**Response:** Revised

Line 191: Why mention it in the abstract if it was excluded?
**Response:** Abstract and materials and method revised. Nursing behavior has traditionally been used as a common behavior to evaluate pain states in piglets. The authors felt it important to acknowledge nursing was evaluated and included in the assessment but is not considered a reliable behavior to assess as an item in the pain scale.

Line 200: Insert the relevant sums before and after castration
**Response:** The authors are unclear what the request is here and it appears number lines are not matching up.

Line 207: Unusual to have citations in a table legend. Also, why use the term grouped moments for time points in the analysis. They are not moments after all but 4 min segments unless I missed something.

**Response:** Citations have been moved to superscripts of the table. The term “moment” has been used to maintain consistent terminology from previous published work on the pain scale.

Line 213: You mean 'in' Table 1

**Response**: Corrected

Line 279: Should this not be the other way round in terms of what is bold or not.

Response: Legend revised to provide better clarity

Line 312: How moderate to intense?

**Response:** There is no current mathematical model to differentiate moderate to intense. For the purposes of this study we defined intense as pain expressed immediately post-castration (M2) and moderate as pain expressed 24 hour post-castration (M4). The manuscript has been updated to reflect this.

Line 313: Therefore, the rescue analgesia protocol did not work?
**Response:** Correct. Sentence revised

Line 348: Tables 7 and 8 are difficult to interpret. Add a description in the legends as to the meaning of one or 2 table items. What does the symbol d signify, for example when only a,b,c are mentioned.
**Response**: Superscripts for all figures and tables have been revised

Line 399: I would prefer the use of the term time(s) rather than moments throughout.
**Response:** Thank you for your comments. I agree that the use of different acronyms may be more intuitive, recording periods have been named to maintain consistency with previous work on different species-pain scales to allow for more effective comparison of the work. The authors choose to keep the recording period names

Line 414: Too much discussion devoted to why nursing was excluded.
**Response:** Discussion section on nursing revised

Line 420: increased

**Response:** Discussion revised

Line 431: Insert ‘to’ after behaviour

**Response:** Discussion revised

Line 439: The fact that there is no chance of detecting pain in sleeping piglets is rather obvious. I am not sure why you bothered to include a sleeping version of the scale and test it so formally.
**Response**: Limitation section revised

Line 449: Yes, so why choose the same 4-minute period from every video? A properly randomised scan sampling analysis would be more appropriate.

**Response:** The authors agree and have provided further explanation on why randomly selected scan samples are difficult to conduct when piglet activity time periods are so short. This has been further added to the limitation section.

Line 451: Grammar: behavioural changes cannot n themselves be expressive. redraft this sentence

Line 453: change ‘was’ to were

Line 456: So in future what are you planning to do about it?
Line 480: insert period punctuation before ‘training’Line 486-489: Very long sentence
Line 497: Delete ‘the’ before human

**Response:** thank you for your thorough review of the discussion. We identify that the discussion was written in a way that was not clear and opportunities were missed to further explain some of the limitations with video selection and rescue analgesic. The discussion has been thoroughly revised, and we appreciate the guidance in making this manuscript more succinct

Reviewer #2: The manuscript describes a thorough analysis of the validity of the UNESP-Botucatu pig composite acute pain scale (UPAPS). Although the statistical analysis is rather comprehensive, the conclusions are limited by major flaws in the experimental design (i.e., a small of group of female piglets as control). These limitations cannot be fixed by text revisions or changes in the statistical analysis and may lead to unreliable conclusions regarding the pain scale, which could negatively impact the evaluation of piglet pain following castration. In addition, the method of castration (tearing of the spermatic cords) is painful and often contraindicated as compared to cutting of the cords. This choice is not justified nor explained in the manuscript, which poses an ethical problem.

If the manuscript is still considered for publication, the design limitations must be rightfully addressed, limitations clearly stated in the discussion, and conclusions nuanced. Further studies are required to validate this scale for piglet castration, and the conclusions should more clearly indicate so. Additional comments regarding specific sections of the manuscript include:

General:
- The whole text needs read-proofing and English check
- Very good use of illustration/video

Abstract:
- Some basic description of the pain scale (general elements recorded) is needed for comprehension

**Response:** Abstract revised

Introduction:
- ¨only 5% of the males piglets in some European countries are castrated with pain relief¨: it should be mentioned that this information is 6 years old. More recent statistics may be different – overall, pain relief is mandatory in many European countries, so this is a bit misleading.

**Response**: Statement clarified to prevent misleading.

Methodology:
- Power calculations are stated, but no justification is given for the lower number of control piglets included. This is a risk of self-replication. In addition, when considering only piglets awake, this sham groups falls to n=6, which is relatively low

**Response:** Thank you for your comments. The term “control” was not an accurate description of the role of the female piglets enrolled on the study and has been changed throughout the manuscript. Given variation in pain sensitivity, the study was designed to permit male piglets to serve as their own control by collecting pain scale data prior to a painful event and collecting data during intense, moderate and mild pain states. The female non-painful piglets were enrolled on the study solely to take into account the effect of natural behavioral variation by day on pain scale results. Previous work published by two co-authors on this manuscript noted behavioral variations by day in castrated horses unassociated with painful states. This work conducted by Trindade and colleagues (2021) noted that pain-related behaviors of horses such as walking and looking out the window decreased, while behaviors such as resting, standing still, and resting pelvic limb increased during the night in comparison with morning or afternoon. Thus, naturally behaviors change throughout the day and providing a non-painful group of female piglets allowed us to take into account such variation in day and time effect on pain scale behavior.

Trindade, P. H. E., Taffarel, M. O., & Luna, S. P. L. (2021). Spontaneous behaviors of post-orchiectomy pain in horses regardless of the effects of time of day, anesthesia, and analgesia. *Animals*, *11*(6), 1629.

- Number of piglet per group analyzed is missing in Table 2, yet very important

**Response:** Piglet number per group has been included in the superscript of the table

- A description of the repartition of the tested piglets within litters is missing
- Litter effects are lacking as random effect in the statistical models (e.g., in the responsiveness and construct validity analysis)

**Response:** Original statistical analysis did not include litter as an effect. Analysis was rerun to include litter as a random effect in the model for responsiveness to control for this effect in our sampling. The addition of litter as a random effect resulted in minimal changes that have been addressed in the results and Tables 7, 8, and 9.

- A complete and detailed ethical statement is missing. It is hard to understand why castration involved tearing of the spermatic cords, shown to be painful, and generally not recommended, rather than cutting. Incisions are also relatively large compared to standard practice.

**Response:** All male piglets located at this facility are required to undergo castration prior to weaning utilizing the approved farm Standard operating procedure and training protocols of the staff.

It is also not clear from the text why analgesia was not administered (if it is because pain was needed to induce for the sake of the study, or because the study aimed at resembling practice, this must be more clearly explained).

**Response**: Methodology section revised to clarify lack of analgesic use

- It is unclear why only a 4-minute clip was used rather than a longer one. Considering that some of the behaviours are short-lasting or only observed at certain points in time (e.g. nursing), this choice can be limiting. Further justification is needed.

**Response:** 4-min clips were selected based on previous work conducted in pain scale research in livestock (Luna et al, 2020). Additionally, one objective of this work is to identify alternative methodologies to quantify pain sensitivity while minimizing labor, equipment, and time requirements for observing behavior. Thus, the selection of 4-min was also conducted to evaluate if pig pain can be detected in shorter behavior observations timepoints

- Some items of the scale deserve a more detailed description, with a more precise choice of words. E.g., how is discomfort observed? Why is quietness part of the posture scoring, are vocalizations taken into consideration? Overall, some of the categories are rather subjective, and deserve more explanation and critical discussion.

**Response:** Scale has been revised to include additional information on each behavioral item

- The justification of why nursing was not retained in the analysis must not only be present in Table 1 but also in the materials & methods / results section.

**Response:** Removal of nursing behavior is stated in results section 295-299.

- The section on distribution of scores could be stronger with some actual statistical testing, rather than descriptive statistics. These should include litter effects.

**Response**: Original statistical analysis did not include litter as an effect. Analysis was rerun to include litter as a random effect in the model for responsiveness to control for this effect in our sampling. The addition of litter as a random effect resulted in minimal changes that haven been addressed in the results and Tables 7, 8, and 9. The distribution of scores serves as a visual representation of the data for readers and is not intended to provide statistical meaning to the manuscript. The remaining statistical analyses presented in the paper serve that role. In addition, the authors added Horn’s parallel analysis and McDonald’s omega coefficient to the statistical package and these tests have been described further in the materials and methods.

- In the multiple association section (PCA results), the ellipses are good graphical representations, but may not be enough to conclude on “on effect of pain”. Statistical models including PC result and moment (with litter as random effect) can support these claims in a more accurate way.

**Response:** Please see previous comments

- It is unclear why specificity is only recorded in M1 and sensitivity only in M2. Why not look at these elements across the 4 recording time points? Further justification is needed.

**Response:** Specificity and sensitivity were only evaluated at two moments as these moments represented the only moments that were identified as 1) non-painful (M1) and 2) painful (M2) given the occurrence or no occurrence of the castration procedure. Calculating the specificity and sensitivity of the other moments is impossible given the pain states varies and intensity of pain is inconsistent.

Discussion:
- It is problematic that all shams were female. This is discussed a bit, but the discussion fails to consider potential cofounding effects (e.g., in terms of social interaction, see Exploration of early social behaviors and social styles in relation to individual characteristics in suckling piglets by Clouard et al)
- I miss a discussion on the scale. Castration-related behaviours observed in past studies include e.g. huddling up or shivering. Why were these not included?
- The discussion on nursing behaviour as indicator of pain lacks a critical reflection on the methods. The fact that nursing was not relevant in this study may be due to the fact that it is not an appropriate indicator of pain, yes, but it can also be due to the study in itself: only 4 minutes clip, while nursing happens approx. 1 or 2 times per hour + nursing is a social behaviour that partly depends on the littermates.
- The discussion on using presence of certain behaviour rather than occurrence frequency deserves more attention. Looking at presence may be more effective, but it may also very much be less accurate (that may be why scratching did not increase in this study). This must be more clearly explained.

- In general, although results are promising, the discussion should be more critical regarding the method: larger-scale studies are still needed (including more piglets, across a wide age range), and the pain scale should also be validated externally by comparison to other quantitative methods to be able to conclude on sensitivity and reliability. Additional, there is lack of validation provided for the pain scale used in the older piglets.

**Response:** Thank you very much for the comments. We appreciate your constructive feedback and agree that there is opportunity for our team to revise the discussion to address the concerns you have noted. We have clarified the role of the non-painful female piglets. These piglets do not serve as the true control for the castrated piglets. Given individual variation in pain sensitivity, the experiment was designed to allow male piglets to serve as their own control, thus assessing and comparing pain scores prior to and following a painful event. The female non-painful piglets were enrolled on the study solely to take into account the effect of natural behavioral variation by day on pain scale results. Previous work published by two co-authors on this manuscript noted behavioral variations by day in castrated horses unassociated with painful states. This work conducted by Trindade and colleagues (2021) noted that pain-related behaviors of horses such as walking and looking out the window decreased, while behaviors such as resting, standing still, and resting pelvic limb increased during the night in comparison with morning or afternoon. Thus, naturally behaviors change throughout the day and providing a non-painful group of female piglets allowed us to take into account such variation in day and time effect on pain scale behavior.
