# Supplementary material for: Validation of the Unesp-Botucatu pig composite acute pain scale (UPAPS) in piglets undergoing castration
Source: PLoS One. 2023 Apr 13;18(4):e0284218. doi: 10.1371/journal.pone.0284218 (PMC10101451; doi:10.1371/journal.pone.0284218)
Supplement: Attachment — Submitted filename: Reviewer responses.docx [file pone.0284218.s012.docx]

Reviewer #1: In my opinion the authors have adequately addressed my queries, but I feel they have not fully replied to the comments of Reviewer 2, but I leave that up to reviewer 2 to decide.

Line 384: change decrease and increase to decreased and increased

**Response**: Updated

Line 413: change 'The UPAPS' to 'the UPAPS'

**Response:** updated

Reviewer #2: I would like to congratulate the authors on a thorough work on improving the article.

The role of the female piglets and use of males as their own controls is now much clearer, and most of the comments on the statistical analysis and content of the article have been addressed.

I still, however, miss an important discussion regarding the content of the scale and the distribution of the behaviours. Although this aspect may have been addressed in previous work, it is central to the discussion, and should be rightfully acknowledged. In particular, I have doubts regarding the pertinence of using the presence of one or more ‘miscellaneous’ behaviours to reach a score for that category. This method implies that behaviours A, B, C or D have a comparable importance for pain recording, and does not take into consideration their intensity or repetition. For instance, prostration may be a stronger sign of pain than biting the bars or objects, and the duration of prostration (4 continuous minutes vs. 2 sec of the behaviour) may also be of importance. Yet these elements are not considered by the scale. The same goes for the ‘attention to the affected area’ category.

For more insights over the relative importance of different behaviours for pain recording in the context of piglet castration, I suggest to read the review ‘Optimal methods of documenting analgesic efficacy in neonatal piglets undergoing castration’ by Sheil and Polkinghorne.

I would like this discussion addressed, as it may affect the reliability and specificity of the scale. In that regard, the scale should be validated by quantitative data.

This element is of importance considering that you have social behaviours in the scale. If all males were castrated, and therefore potentially in pain or disturbed, and all females were handled, therefore potentially stressed, can you really say anything about the interaction of a specific individual with its littermates? Again, this warrants a discussion on the behaviours included in the scale.

**Response:** Thank you for your insight and reference to the article. We have updated the discussion to include limitations to the pain scale and address the impact of social behavior and behavior frequency and appreciate your constructive feedback to improve the discussion.

I am also missing, in the materials and method, an explanation of the distribution of males per litter. Was there a single tested male per litter? 2, 3, 4?

**Response:** information added in materials and methods

Lastly, the dataset used to perform the analysis should be made available.

**Response:** Included
