# Supplementary material for: Validation of the Unesp-Botucatu pig composite acute pain scale (UPAPS) in piglets undergoing castration
Source: PLoS One. 2023 Apr 13;18(4):e0284218. doi: 10.1371/journal.pone.0284218 (PMC10101451; doi:10.1371/journal.pone.0284218)
Supplement: Attachment — Submitted filename: Response to reviewers.docx [file pone.0284218.s013.docx]

Minor language issues:

line 57 instead of "pharmaceutical therapies" say "pharmaceutical pain control" as this is what is important in the context

**Response: changed**

line 99 change to "All male pigs in each litter were enrolled in the study"

**Response: added**

line 149 "and one, four-minute video clips" is not understandable - something missing?

**Response: revised**

line 117 by saying that the sows had ad libitum access to one nipple and one feeder, you say they have access to the devices but you don't actually say anything about their access to water and feed!

**Response: revised**

line 120 "one-trained" should be "one trained"

**Response: changed**

line 122 please be specific as to whether the piglets were suspended by their hindlegs or placed with horizontal support

**Response: revised**

line 163 vs line 187 and any other place in the manuscript where you refer to specific tasks carried out by specific persons: be consistent in how you refer to this and to their identity. Ideally something like "by two observers (initialsA, initialsB)" or for 187 "by the same person (PHET)"

**Response: Added**

line 346 change to "is a critical welfare issue and pain negatively impacts"

**Response: revised**

line 363 "is often difficult to capture on video" - it doesn't seem to be the video that is the issue but the short time frame that you are using for the analysis, as you identify on lines 365-366. Please revise accordingly.

**Response: revised**

line 421 "subtly", not "subtlety"

**Response: Changed**

line 425-26 Please change to "Male piglet number per litter in this study varied".

**Response: changed**
